# Increased Prevalence of Accidents and Injuries in Congenital Adrenal Hyperplasia: A Population-based Cohort Study

**DOI:** 10.1210/clinem/dgad624

**Published:** 2023-10-20

**Authors:** Henrik Falhammar, Angelica Lindén Hirschberg, Agneta Nordenskjöld, Henrik Larsson, Anna Nordenström

**Affiliations:** Department of Endocrinology, Karolinska University Hospital, SE-171 76 Stockholm, Sweden; Department of Molecular Medicine and Surgery, Karolinska Institutet, SE-171 76 Stockholm, Sweden; Department of Women's and Children's Health, Karolinska Institutet, SE-171 76 Stockholm, Sweden; Department of Gynecology and Reproductive Medicine, Karolinska University Hospital, SE-171 76 Stockholm, Sweden; Department of Women's and Children's Health, Karolinska Institutet, SE-171 76 Stockholm, Sweden; Center for Molecular Medicine, Karolinska Institutet, SE-171 76 Stockholm, Sweden; Pediatric Surgery, Astrid Lindgren Children's Hospital, Karolinska University Hospital, SE-171 76 Stockholm, Sweden; School of Medical Sciences, Örebro University, SE-702 81 Örebro, Sweden; Department of Medical Epidemiology and Biostatistics, Karolinska Institutet, SE-171 65 Stockholm, Sweden; Department of Women's and Children's Health, Karolinska Institutet, SE-171 76 Stockholm, Sweden; Department of Pediatric Endocrinology, Astrid Lindgren Children's Hospital, Karolinska University Hospital, SE-171 76 Stockholm, Sweden

**Keywords:** 21-hydroxylase deficiency, trauma, fall, androgen concentrations

## Abstract

**Context:**

It has been suggested that injuries and accidents are increased in females with congenital adrenal hyperplasia (CAH), but the prevalence is unclear.

**Objective:**

To study the prevalence of injuries and accidents in females and males with CAH.

**Design, Setting, and Participants:**

Patients with CAH (n = 714, all 21-hydroxylase deficiency) were compared with matched controls (n = 71 400). Data were derived by linking National Population-Based Registers.

**Main Outcome Measures:**

Prevalence of injuries and accidents.

**Results:**

Mean age was 29.8 ± 18.4 years. Injuries were more prevalent in patients with CAH than in controls (relative risk, 1.34; 95% CI, 1.24-1.44), and this was found in both sexes (females: 1.43; 1.29-1.58; males: 1.25; 1.12-1.38). In the classical phenotype, the prevalence of injuries was higher, especially in females but not in the nonclassic phenotype. In the genotype groups, injuries were mainly increased in females. Head injuries were increased in all patients with CAH and in the different phenotypes and were mainly driven by females. More patients with CAH born before the introduction of neonatal screening had had an injury compared with controls (1.48; 1.35-1.62); this was seen in both sexes. In patients with CAH born after the introduction of screening, the prevalence of injuries was overall increased (1.20; 1.07-1.35), and in females with CAH but not in males. Accidents showed a similar pattern to injuries in all comparisons.

**Conclusion:**

Patients with CAH had an increased prevalence of both injuries and accidents, especially in females and in those born before the neonatal screening program. Patients with nonclassic phenotype were hardly affected.

Congenital adrenal hyperplasia (CAH) is a group of rare autosomal recessive disorders leading to impaired cortisol production from the adrenal cortex (1). The most common form of CAH, 21-hydroxylase deficiency (21OHD), which is caused by pathological variants in the *CYP21A2* gene, affects 95% to 99% of all CAH cases (2, 3). 21OHD results in cortisol and variable aldosterone deficiency together with increased production of steroid precursors and adrenal androgens (1).

CAH can be classified as classic (ie, salt-wasting [SW] and simple virilizing [SV] CAH) and nonclassic (NC) CAH depending on the degree of enzyme deficiency (1). The SW phenotype is fatal if glucocorticoid replacement is not initiated in the neonatal period. Depending on the fetal concentrations of adrenal androgens, girls with classic CAH are born with varying degrees of virilization of the external genitalia, which led to early diagnosis before the introduction of neonatal screening, whereas boys were typically diagnosed because of SW crisis in the SW CAH or rapid postnatal growth and virilization in the SV CAH (3). Neonatal screening for 21OHD has been introduced in many countries, which has led to diagnosis of classic CAH within the first few days of life (3, 4). The NC phenotype is often not detected by the neonatal screening because it results in almost normal cortisol production with milder adrenal androgen excess. NC CAH is typically diagnosed during the teenage or young adulthood period, especially in females (5).

The principal treatment in CAH consists of glucocorticoid replacement and, in classic cases, mineralocorticoid replacement, which makes survival possible in SW CAH and normalizes adrenal androgen production (2, 3). However, glucocorticoid replacement is often supraphysiological, leading to low adrenal androgen concentrations (6-8) and long-term negative cardiometabolic and bone outcomes (9). Importantly, mortality is increased, mainly because of adrenal crisis (10).

Bone mineral density was found to be decreased in a meta-analysis of adults with CAH compared with matched controls (11). The slightly low bone mineral density has been 1 explanation for the increased fracture rate found in patients with glucocorticoid replacement in general (12), and in CAH specifically (13, 14). However, females with CAH have a greater interest in motorsport and other male-dominant hobbies as well as working more often in male-dominant occupations as a result of the effects of increased androgen exposure during fetal life (15). This may be another reason for more fractures in females with CAH compared with matched controls. Moreover, epinephrine production is impaired in classic CAH (16, 17), and this may lead to hypoglycemia and orthostatism, which may increase the risk of falls. Yet, it has not been studied if patients with CAH are exposed to more injuries and accidents compared with the general population.

Thus, the aim of this study was to examine how common injuries and accidents were in patients with 21OHD in Sweden and compare them with matched controls. In addition, differences in frequency between the sexes, different pheno- and genotypes, and before and after the introduction of the nationwide neonatal screening (to see if early diagnosis improved outcomes) were investigated.

## Subjects and Methods

### Subjects

All individuals with 21OHD identified with a complete personal identification number found in the National CAH Registry (n = 640, all born between 1910 and 2013) were selected (2). Moreover, additional patients with CAH found in the National Patient Register (NPR), using the International Classification of Diseases (ICD), ICD-8 (255.01, 255.08), ICD-9 (2552, 255C), and ICD-10 (E25.0), were identified (if registered with these codes at least 3 times) and further scrutinization of all registered ICD codes were performed to make sure the CAH diagnosis was correct. By this procedure, another 74 patients with CAH were identified. The National CAH Registry includes all patients with 21OHD found via the National Neonatal Screening Program, late-diagnosed patients reported to the screening laboratory, all Swedish patients that had had a *CYP21A2* gene analysis performed, and all patients known to our hospital through previous or current clinical contacts or studies (since the 1940s). This process has been reported in detail in numerous previous studies (10, 13, 18-22).

In the majority of patients, both phenotype and genotype were available. Thus, patients with CAH were divided into the 3 phenotype groups, (ie, SW, SV, and NC), along with the 5 most frequent genotype groups (ie, null, I2 splice, I172N, P30L, and V281L) according to *CYP21A2* gene analysis, as previously described (2, 23). The mildest variant defines the genotype group in compound heterozygotes. Null is associated with the SW phenotype, I2 splice is usually associated with SW, I172N with SV, and V281L with NC form (23), whereas the variant P30L is clinically between SV and NC in severity (24) but was defined as SV in the current study. All patients clinically and biochemically and/or genetically verified NC CAH were grouped together in the NC group. The introduction of neonatal screening for 21OHD in Sweden was in 1986; patients born before or after the introduction were identified.

### Study Protocol

In the total Population Register, 100 controls matched for birth year, sex, and place of birth were identified for each case with 21OHD to be able to evaluate rare events. Thus, when a patient with 21OHD was found, 100 controls born the same year (as close as possible to the same date), with the same sex and born in the same municipality were identified. This means that some controls probably were born at the same maternity clinic and even at the same day as the patient. The Migration Records (Statistics Sweden), which include all migrations since 1901, was used to match for migration. The unique Swedish personal identification number made unambiguous linkage possible between the population-based registers. Before delivery, all data were deidentified by the register holders. By using the NPR (Swedish Board of Health and Welfare), all discharge diagnoses according to the ICD for both in- and outpatient specialist care since 1964 and 2001, respectively, were identified. The outcomes, injuries, and accidents were recorded in the study. Transport accidents and falls were also recorded; those data have also been presented in a previous study (13). The prevalence was calculated at the time point of the data extraction (2013), at the time of death, or migration out of the country, whichever came first. The different ICD codes (Swedish version) used for the separate analyses are shown in Table 1.

**Table 1.** Injuries, including fractures, and accidents^a^ based on International Classification of Diseases (ICD) diagnoses from the National Patient Registry including both in- and outpatient care

Diagnosis	ICD8	ICD9	ICD10
Any injury	800-959	800-959	S00-T14
Head injuries	800-804 850-854 830 870-873 910 920 921	800-804 850-854 830 848A 848B 870-873 910 918 920 921 925	S00-S09
Intracranial injury	850-854	850-854	S06
Concussion	850	850	S06.0
Any accidents*^a^*	E807-E846 E880-E949	E800-E849 E880-E929	V01-X39
Number of accidents*^a^* ≥2	E807-E846 E880-E949	E800-E849 E880-E929	V01-X39
Number of accidents*^a^* ≥6	E807-E846 E880-E949	E800-E849 E880-E929	V01-X39
Transport accidents	E807-E846	E800-E849	V01-V99
Pedestrian injured in transport accident	E 807.2	E819H	V01-V09
Motorcycle rider injured in transport accident	E 819.2*, E 819.3*	E819C E819D	V20-V29
Car occupant injured in transport accident	E 819.0*, E 819.1*	E819A E819B	V40-V49
Falls	E880-E887	E880-E888	W00-W19
Fall on same level	E885.9, E 886.9	E885 E886	W00 W01 W03 W18

^*a*^Excludes poisoning and other adverse effects.

The study was approved by the Swedish Ethical Review Authority, Sweden (Dnr 2010/1869-31/11).

### Statistical Analysis

For continuous variables, mean ± SD was used if normally distributed; otherwise, median and range were used. For categorical variables, absolute and relative frequencies were used. Categorical parameters were compared using Fisher exact test and relative risk (RR) calculations with 95% CI for the composite outcome “any injury” and “any accident” in the entire cohort as well as in subgroups. A *P* <.05 was considered statistically significant.

## Results

### Characteristics of Cases and Controls

In total, 714 individuals with 21OHD were included in the study and the mean age was 29.8 ± 18.4 years, with the oldest aged being 83 years. Females with 21OHD (n = 404) had a mean age of 30.5 ± 18.0 years, whereas males with 21OHD (n = 310) had a mean age 28.7 ± 18.8 years (Table 2). In 79.3% of patients with 21OHD (n = 566), the severity of the disease was available. Table 3 displays the specific details concerning the number of patients with 21OHD in the different phenotype groups (SW, n = 288; SV, n = 188; and NC, n = 90, respectively) and their mean ages. Table 4 displays the different genotype groups (null, n = 115; I2 splice, n = 155; I172N, n = 146; and P30L, n = 29, respectively) and their mean ages. Of the patients with 21OHD, 355 (females, n = 197) were born before the introduction of the Swedish national neonatal screening program for 21OHD in 1986 (4), and 379 (females, n = 207) thereafter (Table 5). Sex, year, and place of birth matched controls were included from the Total Population Registry (n = 71 400). This cohort of patients with 21OHD was updated up to 2013 (previously 2009) (13, 21, 22, 25), including more patients than in the cohort of patients with 21OHD reported earlier by our group in prior studies (10, 18-20).

**Table 2.** Injuries and accidents^a^ in individuals with congenital adrenal hyperplasia (CAH) resulting from 21-hydroxylase deficiency, also divided into females and males, compared with age- and sex-matched controls (100 controls per case)

	CAH individuals	Controls	*P*	CAH females	Controls females	*P*	CAH males	Controls males	*P*
n	714	71 400		404	40 400		310	31 000	
Any injury	362 (50.7%)	27 093 (37.9%)	**<**.**00001**	195 (48.3%)	13 679 (23.9%)	**<**.**00001**	167 (53.9%)	13 414 (43.3%)	.**0002**
Relative risk (95% CI)	1.34 (1.24-1.44)			1.43 (1.29-1.58)			1.25 (1.12-1.38)		
Head injury	133 (18.6%)	9738 (13.6%)	.**0002**	68 (16.8%)	4346 (10.8%)	.**0003**	65 (21.0%)	5392 (17.4%)	.1136
Intracranial	61 (8.5%)	4079 (5.7%)	.**0013**	34 (8.4%)	1967 (4.9%)	.**0012**	27 (8.7%)	2112 (6.8%)	.1891
Concussion	56 (7.8%)	3790 (5.3%)	.**0029**	32 (7.9%)	1852 (4.6%)	.**0038**	24 (7.7%)	1938 (6.3%)	.2876
Any accidents*^a^*	289 (40.5%)	21 678 (30.4%)	**<**.**00001**	160 (39.6%)	10 925 (26.6%)	**<**.**00001**	129 (41.6%)	10 753(32.9%)	.**0118**
RR (95% CI,)	1.33 (1.22-1.46)			1.46 (1.30-1.65)			1.20 (1.05-1.37)		
≥2	156 (21.8%)	10 711 (15%)	**<**.**00001**	81 (20%)	5050 (12.5%)	**<**.**00001**	75 (24.2%)	5 661 (18.3%)	.**0096**
≥6	32 (4.5%)	1 650 (2.6%)	.**0006**	15 (3.7%)	673 (1.7%)	.**005**	17 (5.5%)	977 (3.2%)	.**0317**
Transport accidents	56 (7.8%)	4244 (5.9%)	.**0385**	34 (8.4%)	2204 (5.5%)	.**015**	22 (7.1%)	2040 (6.6%)	.7292
Pedestrian	0 (0%)	169 (0.2%)	.4209	0 (0%)	90 (0.2%)	1	0 (0%)	79 (0.3%)	1
Motorcycle rider	7 (1.0%)	725 (1.0%)	1	2 (0.5%)	198 (0.5%)	.7266	5 (1.6%)	527 (1.7%)	1
Car occupant	26 (3.6%)	138 2(1.9%)	.**0025**	14 (3.5%)	756 (1.9%)	.**0264**	12 (3.9%)	626 (2.0%)	.**0385**
Any falls	190 (26.6%)	13 722(19.2%)	**<**.**00001**	102 (25.3%)	7118 (17.6%)	.**0001**	88 (28.4%)	6604 (21.3%)	.**0034**
Fall on same level	111 (15.6%)	706 5(9.9%)	**<**.**00001**	59 (14.6%)	3671 (9.1%)	.**0003**	52 (16.8%)	3394 (11.0%)	.**0019**

Bold type indicates *P* < .05.

^*a*^Excludes poisoning and other adverse effects.

**Table 3.** Injuries and accidents^a^ in individuals with CAH because of 21-hydroxylase deficiency divided into the phenotypes and compared with age- and sex-matched controls (100 controls per case)

	SW			SV			NC		
	All	Females	Males	All	Females	Males	All	Females	Males
n	288	157	131	188	101	87	90	67	23
Mean age ± SD (y)	24.5 ± 16.1	25.3 ± 15.6	23.6 ± 16.7	32.5 ± 19.3	32.2 ± 17.5	32.8 ± 21.3	29.3 ± 15.9	30.2 ± 15.7	26.7 ± 16.4
Any injury	**143** (49.7%)***	**79** (50.3%)***	**64** (48.9%)*	**104** (55.3%)***	**55** (54.5%)***	**49** (56.3%)*	*41 (45.6%)* ^#^	*29 (43.3%)* ^#^	12 (52.2%)
RR (95% CI,)	**1.33** (**1.18-1.49)**	**1.46** (**1.25-1.71)**	**1.19** (**1.00-1.42)**	**1.46** (**1.29-1.67)**	**1.69** (**1.41-2.03)**	**1.27** (**1.05-1.53)**	*1.24* (*0.99-1.56)*	*1.31* (*0.99-1.73)*	1.11 (0.75-1.65)
Head injury	**52** (18.1%)*	**28** (17.8%)*	24 (18.3%)	**38** (20.2%)**	**18** (17.8%)*	20 (23%)	**19** (21.1%)*	**13** (19.4%)*	6 (26.1%)
Intracranial injury	22 (7.6%)	*13 (8.3%)* ^#^	9 (6.9%)	**21** (11.2%)**	**12** (11.9%)**	9 (10.3%)	7 (7.8%)	5 (7.5%)	2 (8.7%)
Concussion	21 (7.3%)	*13 (8.3%)^#^*	8 (6.1%)	**20** (10.6%)**	**12** (11.9%)**	8 (9.2%)	7 (7.8%)	5 (7.5%)	2 (8.7%)
Any accidents*^a^*	**114** (39.6%)**	**64** (40.8%)***	50 (38.2%)	**87** (46.3%)***	**47** (46.5%)***	**40** (46.0%)*	32 (35.6%)	24 (35.8%)	8 (34.8%)
RR (95% CI,)	**1.30** (**1.13-1.51)**	**1.45** (**1.20-1.75)**	1.15 (0.93-1.44)	**1.56** (**1.34-1.82)**	**1.85** (**1.50-2.29)**	**1.31** (**1.04-1.64)**	1.17 (0.88-1.54)	1.32 (0.95-1.82)	0.87 (0.49-1.52)
≥2	**62** (21.5%)**	**30** (19.1%)*	**32** (24.4%)*	**46** (24.5%)***	**24** (23.8%)**	22 (25.3%)	12 (13.3%)	10 (14.9%)	2 (8.7%)
≥6	**10** (3.5%)**	5 (3.2%)	5 (3.8%)	**12** (6.4%)**	**6** (5.9%)**	6 (6.9%)	2 (5.3%)	2 (3%)	
Transport accidents	2 2(7.6%)	12 (7.6%)	10 (7.6%)	16 (8.5%)	**12** (11.9%)*	4 (4.6%)	4 (4.4%)	3 (4.5%)	1 (4.4%)
Motorcycle rider	2 (0.7%)	1 (0.6%)	1 (0.8%)	2 (1.1%)	1 (1%)	1 (1.2%)	1 (1.1%)		1 (4.4%)
Car occupant	**13** (4.5%)**	**7** (4.5%)*	**6** (4.6%)*	6 (3.2%)	4 (4%)	2 (2.3%)			
Any falls	**73** (25.4%)**	**43** (27.4%)**	30 (22.9%)	**62** (33%)***	**30** (29.7%)***	**32** (36.8%)**	22 (22.2%)	15 (22.4%)	5 (21.7%)
Fall on same level	**44** (15.3%)**	**25** (15.9%)**	19 (14.5%)	**33** (17.6%)**	**16** (15.8%)*	**1 7**(19.5%)*	10 (11.1%)	7 (10.5%)	3 (13%)

Abbreviations: CAH, congenital adrenal hyperplasia; RR, relative risk.

Bold indicates *P* < .05. Italic indicates *P* = .05–.09. Numbers are not displayed if no patient had the condition.

^*a*^Excludes poisoning and other adverse effects.

^*^ *P* < .05.

^**^ *P* < .01.

^***^ *P* < .001.

^#^ *P* = .05-.099.

**Table 4.** Injuries, including fractures, and accidents^a^ in individuals with CAH constituting the four most common *CYP21A2* genotype groups compared with age- and sex-matched controls (100 controls per case); severity of the genotype ranges from left to right

	Null			I2 splice			I172N				P30L	
	All	Females	Males	All	Females	Males	All	Females	Males	All	Females	Males
n	115	63	52	155	85	70	146	79	67	29	15	14
Mean age ± SD (y)	23.9 ± 14.8	24.7 ± 13.2	22.9 ± 16.7	23.8 ± 16.7	24.6 ± 17.0	22.7 ± 16.4	32.8 ± 20.4	32.8 ± 18.6	32.7 ± 22.4	25.6 ± 10.3	26.2 ± 10.4	24.9 ± 10.5
Any injury	**65** (56.5%)***	**36** (57.1%)***	**29** (55.8%)*	**68** (43.9%)*	**39** (45.9%)**	29 (41.4%)	**82** (56.2%)***	**45** (57.0%)***	**37** (55.2%)*	**17** (58.6%)*	**7** (46.7%)*	**10** (71.4%)*
RR (95% CI,)	**1.48** (**1.26-1.74)**	**1.53** (**1.23-1.90)**	**1.42** (**1.11-1.81)**	**1.19** (**1.00-1.43)**	**1.41** (**1.12-1.79)**	0.99 (0.74-1.31)	**1.47** (**1.28-1.70)**	**1.71** (**1.41-2.07)**	**1.26** (**1.02-1.57)**	**1.65** (**1.21-2.24)**	**1.94** (**1.12-3.36)**	**1.49** (**1.06-2.08)**
**Head injury**	**25** (21.7%)*	**16** (25.4%)**	9 (17.3%)	22 (14.2%)	12 (14.1%)	10 (14.3%)	*28 (19.2%)* ^#^	12 (15.2%)	16 (23.9%)	6 (20.7%)	**4** (26.7%)*	2 (14.3%)
Intracranial	*11 (9.6%)* ^#^	**9** (14.3%)**	2 (3.9%)	10 (6.5%)	4 (4.7%)	6 (8.6%)	**17** (11.6%)**	**9** (11.4%)*	8 (11.9%)	**4** (13.8%)*	**3** (20%)*	1 (7.1%)
Concussion	**11** (9.6%)*	**9** (14.3%)**	2 (3.9%)	10 (6.5%)	4 (4.7%)	6 (8.6%)	**16** (11%)**	**9** (11.4%)*	7 (10.5%)	**4** (13.8%)*	**3** (20%)*	1 (7.1%)
Any accidents*^a^*	**51** (44.3%)**	**30** (47.6%)*	21 (40.4%)	54 (34.8%)	**30** (35.3%)*	24 (34.3%)	**66** (45.2%)***	**37** (46.8%)***	29 (43.3%)	**14** (48.3%)*	**6** (40%)*	8 (57.1%)
RR (95% CI,)	**1.40** (**1.14-1.72)**	**1.49** (**1.15-1.93)**	1.2 9(0.93-1.80)	1.18 (0.95-1.46)	**1.38** (**1.03- 1.83)**	1.00 (0.72-1.38)	**1.52** (**1.27-1.82)**	**1.82** (**1.43-2.31)**	1.25 (0.95-1.65)	**1.68**(**1.15- 2.45)**	**2.11** (**1.12-3.95)**	1.45 (0.92-2.30)
≥2	**31** (27%)***	**19** (30.2%)**	12 (23.1%)	25 (16.1%)	9 (10.6%)	16 (22.9%)	**37** (25.3%)***	**17** (21.5%)**	**20** (29.9%)*	6 (20.7%)	*4 (26.7%)* ^#^	2 (14.3%)
≥6	4 (3.5%)	3 (4.8%)	1 (1.9%)	6 (3.9%)	2 (2.4%)	4 (5.7%)	**8** (5.5%)*	*3 (3.8%)* ^#^	*5 (7.5%)* ^#^	**3** (10.3%)*	**2** (13.3%)**	1 (7.1%)
Transport accidents	11 (9.6%)	*8 (12.7%)* ^#^	3 (5.8%)	9 (5.8%)	2 (2.4%)	7 (10%)	**15** (10.3%)*	**11** (13.9%)**	4 (6%)	1 (3.5%)	1 (6.7%)	
Motorcycle rider				1 (0.7%)		1 (1.4%)	2 (1.4%)	1 (1.3%)	1 (1.5%)			
Car occupant	**5** (4.4%)*	3 (4.8%)	2 (3.9%)	*6 (3.9%)* ^#^	2 (2.4%)	**4** (5.7%)*	*6 (4.1%)* ^#^	*4 (5.1%)* ^#^	2 (3%)			
Any falls	**34** (29.6%)*	**22** (34.9%)*	12 (23.1%)	33 (21.3%)	19 (22.4%)	14 (20%)	**4 9**(33.6%)***	**24** (30.4%)**	**25** (37.3%)**	**10** (34.5%)*	*4 (26.7%)* ^#^	6 (42.9%)
Fall on same level	**18** (15.7%)*	**12** (19.1%)*	6 (11.5%)	**24** (15.5%)**	**12** (14.1%)*	*12 (17.1%)* ^#^	**25** (17.1%)**	*12 (15.2%)* ^#^	*13 (19.4%)* ^#^	**7** (24.1%)**	**3** (20%)*	*4 (28.6%)* ^#^

Abbreviations: CAH, congenital adrenal hyperplasia; RR, relative risk.

The numbers are not displayed if no patient had the condition, but analysis showed *P* > .10. Bold indicates *P* < .05. Italic indicates *P* = .05-.09.

^*a*^Excludes poisoning and other adverse effects.

^*^ *P* < .05.

^**^ *P* < .01.

^***^ *P* < .001.

^#^ *P* = .05-.09.

**Table 5.** Injuries and accidents in individuals with CAH because of 21-hydroxylase deficiency divided into those born before the introduction of neonatal screening (1986) and those afterwards compared with age- and sex-matched controls (100 controls per case)

	Born before introduction of neonatal screening					Born after introduction of neonatal screening				
	CAH individuals	Controls	*P*	CAH females	CAH males	CAH individuals	Controls	*P*	CAH females	CAH males
n	335	33 500		197	138	379	37 900		207	172
**Any injury**	197 (58.8%)	13 327 (39.8%)	**<.00001**	**109** (57.8%)***	**88** (63.8%)***	165 (43.5%)	13 766 (36.3%)	**.0044**	**86 (41.5%)** *	79 (45.9%)
RR (95% CI,)	**1.48 (1.35-1.62)**			**1.62 (1.43-1.84)**	**1.33** (**1.17-1.51)**	**1.20 (1.07-1.35)**			**1.24** (**1.05-1.46)**	1.16 (0.99-1.37)
**Any accident** *^z^*	148 (44.2%)	9392 (28%)	**<.00001**	**84** (42.6%)***	**64** (39.8%)**	141 (41.2%)	12 286 (40.1%)	.0535	**76 (36.7%)** *	65 (37.8%)
RR (95% CI,)	1.58 (1.40-1.78)			**1.77 (1.50-2.09)**	**1.38** (**1.15-1.65)**	1.15 (1.00-1.31)			**1.23** (**1.03-1.47)**	1.07 (0.88-1.29)

Abbreviations: CAH, congenital adrenal hyperplasia; RR, relative risk.

Bold indicates *P* < .05. Italic indicates *P* = .05-.09.

^<^Excludes poisoning and other adverse effects.

^*^ *P* < .05.

^**^ *P* < .01.

^***^ *P* < .001.

### Any Injury

Injuries were more prevalent in patients with CAH than in controls (50.7% vs 37.9%; RR, 1.34; 95% CI, 1.24-1.44), and for both females and males (females: 48.3% vs 23.9%; RR, 1.43; 95% CI, 1.29-1.58; males: 53.9% vs 43.3%; RR, 1.25; 95% CI, 1.12-1.38) (Table 2). For the phenotype groups SW and SV, the prevalence of any injury was higher than in controls, especially in females (Table 3). Individuals with NC CAH did not demonstrate an increased prevalence of any injury. When analyzing the genotype groups separately, it was mainly the females who had increased injury prevalence compared with controls (Table 4).

### Head Injury

Head injury, including intracranial injury and concussion, was increased in females with CAH but not in males with CAH (Table 2). Increased head injuries in females were also seen when assessing the phenotypes separately (Table 3). Intracranial injury and concussion were only increased in the females with the SV phenotype. Head injury, including intracranial injury and concussion, was increased in females with the null genotype (Table 4). Intracranial injury and concussion were more prevalent in females with I172N and P30L genotypes.

### Any Accidents

Accidents were more prevalent among patients with CAH than controls (40.5% vs 30.4%; RR, 1.33; 95% CI, 1.22-1.46), and in both females and males (females: 39.6% vs 26.6%; RR, 1.46; 95% CI, 1.30-1.65; males: 41.6% vs 32.9%; RR, 1.20; 95% CI, 1.05-1.37) (Table 2). Also, 2 or more accidents and 6 or more accidents were more prevalent in patients with CAH than controls, and similar for both sexes. When analyzing the SW and SV phenotype groups, especially females had more accidents compared with controls, whereas patients with NC CAH did not demonstrate any increased prevalence of accidents (Table 3). When analyzing the genotype groups, the females had more accidents than the males (Table 4).

### Transport Accidents

Transport accident was more common in females with CAH compared with controls (Table 2). These accidents often occurred as a car occupant (ie, driver or passenger). There was no increase in motorbike accidents. When assessing phenotype groups, only females with SV had more transport accidents compared with controls. However, accidents as a car occupant were increased for all patients with SW phenotypes as well as when analyzing women and men separately. In the I172N genotype group, transport accidents were increased in females. Car occupant accidents were increased for all patients with null genotype and males with I2 splice genotype.

### Fall Accidents

Fall accidents were more prevalent in all patients with CAH and both sexes (Table 2). Also, fall on the same level was increased when assessing these groups. In the SW phenotype, any falls and fall on the same level were increased in females (Table 3). In the SV phenotype, both any falls and fall on the same level were increased in both sexes. No increase in falls was seen in patients with the NC phenotype.

Females with null genotype demonstrated more falls and fall on the same level (Table 4). In the I2 splice group, only fall on the same level was increased in females, whereas in the I172N group, both sexes had increased falls. Females with the P30L genotype had more falls on the same level compared with controls.

### Before and After the Introduction of Neonatal Screening

More patients with CAH born before the introduction of neonatal screening had had an injury compared with controls (58.8% vs 39.8%; RR, 1.48; 95% CI, 1.35-1.62); this was also seen in both sexes (Table 5). Similar increases were seen in accidents compared with controls.

In those patients with CAH born after the introduction of neonatal screening, the prevalence of injuries was less increased in all (43.5% vs 36.3%; RR, 1.20; 95% CI, 1.07-1.35) and in females with CAH compared with controls but no significant increase at all was found in males. Accidents were only increased in females with CAH and not in males (Table 5).

### Injuries and Accidents Without Fractures

When all cases and controls in which a fracture had occurred at the time of the injury were excluded, patients with CAH still had more injuries (27.2% vs 21.9%; RR, 1.21; 95% CI, 1.10-1.40), and similarly for females but not males (females: 28.7% vs 20.5%; RR, 1.39; 95% CI, 1.20-1.63; males: 25.2% vs 23.6%; RR, 1.06; 95% CI, 0.88-1.29). More patients with CAH born before the introduction of neonatal screening had had an injury without fracture compared with controls (29.0% vs 22.0%; RR, 1.31; 95% CI, 1.11-1.56) and similarly for females but not males (females: 30.0% vs 16.8%; RR, 1.78; 95% CI, 1.44-2.21; males: 29.0% vs 22.0%; RR, 1.09; 95% CI, 0.83-1.44). After the introduction of screening, there were only more injuries without fractures in females with CAH compared with controls (all: 25.6% vs 21.7%; RR, 1.17; 95% CI, 0.99-1.40; females: 27.5% vs 21.4%; RR, 1.29; 95% CI, 1.03-1.61; males: 23.3% vs 22.2%; RR, 1.05; 95% CI, 0.79-1.38). In patients with SW CAH, only females had more injuries without fractures compared with controls (all: RR, 1.26; 95% CI, 1.05-1.52; females: RR, 1.45; 95% CI, 1.15-1.54; males: RR, 1.05; 95% CI, 0.78-1.43), which was similar in patients with SV CAH (all: RR, 1.25; 95% CI, 0.98-1.58; females: RR, 1.47; 95% CI, 1.08-2.00; males: RR, 1.03; 95% CI, 0.71-1.50), whereas in patients with NC CAH, there were no significant differences compared with controls (all: RR, 1.36; 95% CI, 0.99-1.87; females: RR, 1.43; 95% CI, 0.99-2.08; males: RR, 1.20; 95% CI, 0.64-2-23). Similar relative risk as in all injuries were seen in head injuries without fractures, and the different genotypes (data not shown). Similar findings were also seen in accidents without fractures (data not shown).

## Discussion

This is the first study examining injuries and accidents in patients with CAH, and it included all patients diagnosed with 21OHD in an entire nation. Injuries and accidents were more prevalent in patients with CAH, in both females and males. However, when assessing the pheno- and genotype groups, females seemed more affected. Patients with NC phenotype did not have more injuries (except for head injuries in females) or accidents. Those patients with CAH born before the introduction of neonatal screening appeared to be especially affected, whereas in those born after, only females had a smaller increase.

The relative risk of any injury or any accident were higher in all patients with CAH compared with controls, and this was seen in both sexes, albeit lower in males (RR, 1.43 and 1.25, respectively). Injuries and accidents were more common in patients before the introduction of neonatal screening but not generally in all born after, where only females with CAH to a lesser degree had increased risk. Head injuries were more common in patients with CAH, especially females, whereas the RR for males with CAH did not differ significantly compared with controls. Transport accidents, mainly as a car driver or passenger, or fall generally or on the same level were more frequent in patients with CAH. In the different pheno- and genotypes, both injuries and accidents were more common in females compared with controls, whereas in males, there were almost no significant differences compared with controls. In those with the least severe variant of CAH, the NC phenotype, the frequency of any injury or accident was similar to controls in both sexes. However, head injuries, for unknown reason, were slightly higher in females with NC CAH compared with controls. Injuries and accidents have not been studied before in a similar manner, which makes these findings novel; however, the transport accident and fall data have been presented previously (13).

In fact, injuries and accidents in females with CAH seemed to be more similar to male controls than the female controls. We know that exposure to high androgen concentrations during fetal life in females with classic CAH results in more gender-atypical behavior. Girls with CAH preferred playing with toy cars more than control girls but slightly less than control boys (26). The toy play could also be related to severity of CAH (ie, genotype group) (27). In adult life, women with CAH reported being more interested in sports, rough sports, and motor vehicles. In addition, their professions were more male typical/male dominant than for the controls (15). Interestingly, there were a direct correlation with the severity of the pathological variants and gender-atypical behavior, in play behavior and in interests in adult life, with women with the null genotype being most extreme (15, 27). Moreover, sports activities more than 2 hours a week was more common in women with CAH than in controls (28). It can be hypothesized that women with more gender-atypical behavior are exposed to more trauma, which in turn causes more emergency visits because of injuries and accidents. It can be speculated that postnatal androgen exposure did not, in the current study, affect the frequency of injuries and accidents to the same extent because females with NC phenotype only had increased risk of head injuries but no other injuries or accidents. Having said that, the NC group, especially the males with NC CAH, was limited, resulting in a lower statistical power compared with the classical groups. Moreover, we did not have biochemical data available, so we do not know the precise androgen exposure. Furthermore, many other factors except phenotype may influence postnatal androgen exposure such as timing of diagnosis, treatment administered, degree of ACTH suppression achieved, and individual compliance.

Thus, many of the injuries and accidents may be due to more gender-atypical behavior. However, the injuries and accidents recorded here were from hospital visits. If an injury or accident occurred and did not result in a hospital visit, these would be unknown to us. Moreover, in- and outpatient specialist care were not registered in the NPR until 1964 and 2001, respectively, explaining why the accident rate was lower in the prescreening controls (older patients thus had experienced some accidents in years when data were not captured) than the postscreening controls. Thus, both injuries and accidents in patients and controls in the prescreening group were probably underestimated. Furthermore, the routines for how injuries and accidents were registered may have changed over the years but this affects patients and controls similarly and the RR will be the same. Patients on glucocorticoid replacement may be more prone to seek medical attention after an accident; however, in this study, car accidents were studied, which would not infer a difference. Supraphysiological glucocorticoid replacement may be a contributing factor to fractures and an accident leading to a fracture is more likely to result in an emergency visit than if no fracture occurred. Because patients with CAH were more likely to get a fracture, this partly explained the increased injury and accident rate. However, there was still an increased risk when the occasions where fractures had occurred were removed. Patients with NC CAH also had fewer fractures than patients with more severe forms of CAH (13, 29).

Falls were more common in classic CAH, including falls on the same level. Females with CAH have been reported to have more visual issues of unclear reason (28), and this may increase the risk of stumbling and tripping. Neurological issues (seizures and migraine) are also more common in females with classic CAH (28) and may play a role in falls and injuries. Moreover, because epinephrine production is decreased in the most severe phenotypes (16, 17), this may lead to orthostatism and hypoglycemia, which may also increase the risk of falls and injuries.

Injuries and accidents may also occur because of violence. Testosterone concentrations have been reported to be associated with violence and criminal behavior (30). However, a national population-based study by our group did not find any association with CAH and conviction of any crime, violent crime, or sex crime (31). Interestingly, these convictions were increased in women with polycystic ovary syndrome (ie, a condition with increased concentrations of androgens postnatally) (31). We know that women with CAH and good adherence to medications normally have low androgen concentrations (6, 7), which may explain the differences.

Alcohol and drug misuse are also associated with injuries and accidents (32). Both females and males with classic forms of CAH had increased alcohol and drug misuse in our previous studies (18, 33). This may also contribute to an increased risk for injuries and accidents in patients with classic CAH.

Why injuries and accidents in the present study seemed to be less common among patients with CAH born after the introduction of neonatal screening is unclear. Females with CAH who had been neonatally screened had increased risk of injuries and accidents but not as pronounced as those diagnosed by clinical signs and symptoms before the screening. It could be speculated that a small difference between patients with CAH and controls increases with age. Of note is also that males with CAH that had been diagnosed clinically before the screening had increased risk of injuries and accidents. Increased adrenal androgen concentrations in males with CAH, seen in cases of late diagnosis or poor adherence, can lower total testosterone concentration because of inhibition of gonadotropins (34). Before the introduction of neonatal screening, particularly boys but also some girls with the SV CAH phenotype were diagnosed later during childhood and were therefore not on replacement doses of glucocorticoids from the neonatal period (6, 17). Hypogonadism in males results in decline in muscle mass and strength (35), which may contribute to more injuries and accidents. On the other hand, patients born after the introduction of neonatal screening probably had better management, and this may have led to less injuries and accidents. In addition, fracture rate have been shown to be normalized by neonatal screening, which may be less surprising (13).

We, in a previous registered-based study, could not find any mortality resulting from accidents and injuries, albeit 2 deaths (10%) were due to suicide (10). Suicide attempts have been shown to be increased in Swedish males with CAH and in 1 pan-European study in females with CAH but not in Swedish females with CAH (18, 28, 33), but we did not examine suicide attempts in the present study.

The major limitation of the current study is its dependence on national registries for all outcome data. The injury and accident prevalences are likely underestimated because minor injuries and accidents probably did not result in hospital care and thus were not included in the national patient registries. Furthermore, a requirement for obtaining ethical approval was that all included patients and controls were anonymized to protect their integrity, which is why we were not able to compare the results with data from medical files. Moreover, even though a large number of patients with CAH were included, the numbers, when divided into the different pheno- and genotype groups, were limited, making the results from the subgroup analyses less reliable because of the low sample sizes. In contrast, the strength of the present study is the usage of the unique national CAH registry with extremely high coverage of all patients diagnosed with CAH in the country, with geno- and phenotype data available for most. To make the results more robust, 100 matched controls were included for each patient with CAH.

In conclusion, patients with CAH had an increased prevalence of both injuries and head injuries, including intracranial injury and concussion, as well as accidents. Both sexes were affected, but females differed more compared with their female controls (to a higher degree). Females with classic CAH (ie, SW and SV phenotype) had most injuries and accidents. Females with CAH born after the introduction of neonatal screening had a less pronounced increase and males had no increased rates, which may indicate that this will be less of an issue in the future when all classic patients have been diagnosed neonatally and received modern management.

## Data Availability

Restrictions apply to the availability of data generated or analyzed during this study to preserve patient confidentiality or because they were used under license. The corresponding author will on request detail the restrictions and any conditions under which access to some data may be provided.

## Abbreviations

21OHD: 21-hydroxylase deficiency
CAH: congenital adrenal hyperplasia
ICD: International Classification of Diseases
NC: nonclassic
NPR: National Patient Register
RR: relative risk
SV: simple virilizing
SW: salt wasting
